# Engaging African Americans in developing an intervention to reduce breast cancer recurrence: A brief report

**DOI:** 10.21633/jgpha.6.120

**Published:** 2016

**Authors:** Selina A. Smith, Mary S. Whitehead, Joyce Q. Sheats, Brittney Fontenot, Ernest Alema-Mensah, Benjamin Ansa

**Affiliations:** 1 Institute of Public and Preventive Health, Augusta University, Augusta, GA; 2 Department of Family Medicine, Medical College of Georgia, Augusta University, Augusta, GA; 3 SISTAAH Talk Breast Cancer Support Group, Miami, FL; 4 Department of Community Health and Preventive Medicine, Morehouse School of Medicine, Atlanta, GA

**Keywords:** Community engagement, African Americans, breast cancer survivors, lifestyle intervention, cancer prevention guidelines

## Abstract

**Background:**

To develop a culturally appropriate lifestyle intervention, involvement of its intended users is needed.

**Methods:**

Members of an African American (AA) breast cancer support group participated in two 4-hour guided discussions, which were audiotaped, transcribed, and analyzed to guide the content.

**Results:**

The support group collaborated with researchers to develop 24 experiential nutrition education sessions using a social cognitive framework and incorporating self-regulation skills (goal-setting, self-monitoring, problem-solving, stimulus control) and social support to enhance self-efficacy for changes in dietary intake.

**Conclusions:**

Community engagement fostered autonomy, built collaboration, and enhanced the capacity of AA breast cancer survivors to participate in developing a lifestyle intervention.

## INTRODUCTION

African American (AA) women are less likely to be diagnosed but are more likely to die from breast cancer than White women (American Cancer Society (ACS), 2015). Unhealthy eating and post-treatment weight gain are contributors (McCollough et al., 2011). Nevertheless, AA breast cancer survivors (BCSs) rarely collaborate with researchers to develop lifestyle interventions.

Community engagement is the process of working collaboratively with groups of people affiliated by similar situations to address issues affecting their well-being (Centers for Disease Control and Prevention, 1997). This brief report describes the process of engaging AA BCSs in developing a program for experiential nutrition education.

## METHODS

**Figure 1:**
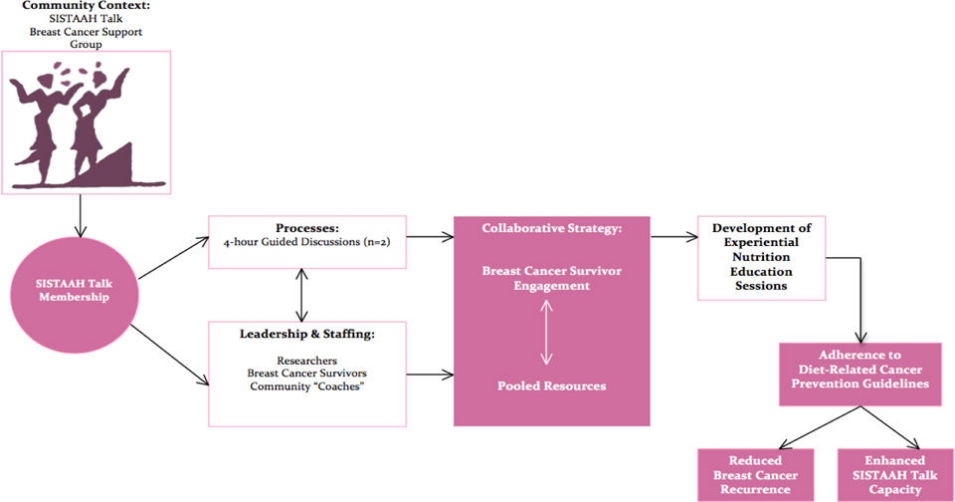
Community engagement process in developing a lifestyle intervention for AA

Researchers engaged members of the SISTAAH (Survivors Involving Supporters to Take Action in Advancing Health) Talk breast cancer support group to develop a dietary intake intervention to enhance the prognoses for BCSs. The community coalition action theory (Butterfoss & Kegler, 2002) was used to develop a flowchart of the community engagement methods employed in this process (Figure 1). The Institutional Review Board of Augusta University approved this study, and participant consent was obtained prior to enrollment.

Established in 1995, SISTAAH Talk has a purpose of providing a forum for AA women to communicate about and make sense of their breast cancer experience in order to achieve improved physical and mental health outcomes. Membership of the support group includes more than 200 survivors. Founded by a breast cancer researcher, SISTAAH Talk is facilitated by an MPH-level BCS and “coaches” or role models trained to lead lifestyle programs.

The two 4-hour guided discussions were led by a BCS trained in qualitative research methods. Each discussion focused on the content and value of the intervention, including cultural appropriateness, comprehension of health messages, length, planned delivery format, likelihood of attendance, and likelihood of recommending it to others. From BCS feedback, interactive, or experiential nutrition education (e.g., cooking demonstrations, label-reading activities, and grocery store tours) was selected as the intervention approach. A review of similar published interventions (Paxton et al., 2011; Demark-Wahnefried et al., 2008) suggested that 24 weeks was the optimal intervention period. Diet-related cancer prevention guidelines (ACS, 2015; World Cancer Research Foundation/American Institute for Cancer Research, 2007) were used to construct the intervention content.

The discussions were digitally recorded, transcribed verbatim, manually coded, and summarized. NVIVO 10 software (2015) was used to facilitate the coding process. Data were analyzed with Qualitative Content Analysis (Schreier, 2012). Recurring themes were identified and summarized.

## RESULTS

For participants (n=60; mean age 45.73 years; SD 7.91; range 35–75 years old), there were two guided discussions, with findings organized into three categories: 1) solving barriers to accessing healthy foods in grocery stores and restaurants; 2) linking behavior change to cultural values; and 3) modifying traditional or favorite dishes to reduce calorie, fat, and sodium content.

BCSs discussed the challenges of locating healthy food in their neighborhoods. One participant stated:

“It can be tough. The closest grocery store is 7 miles away. If I don’t stop by the store on the way home, I am stuck with over-priced processed food from the corner store. I try not to eat out because fast-food places are the only restaurants in my neighborhood.”

In considering the effects of culture on healthy eating, one BCS reflected:

“Take my collards (greens) away from me, and its over. I switched from (adding) fatback to smoked turkey and now I’ve learned that smoked meats cause cancer. What are we supposed to do?”

The need to transform traditional (e.g., soul food, southern dishes, and Caribbean favorites) recipes into healthier versions was discussed. One participant said:

“I am not opposed to eating new foods, but I am never eating kale, even though they call it the new super food. I live in the inner city—and am proud of it. I want to eat the food that I love. I just want to make it healthier.”

The perceptions, strategies, and recommendations of the BCSs guided development of the intervention. Social cognitive theory, incorporating self-regulation skills (goal-setting, self-monitoring, problem-solving, stimulus control) and social support to enhance self-efficacy (Zoellner et al., 2011), undergirded the sessions (Table 1).

## DISCUSSION/CONCLUSIONS

Lifestyle behaviors, such as dietary intake, are involved in the development and recurrence of breast cancer and affect the quality of life for survivors. AA women are less likely to participate in traditional lifestyle modification programs. To meet the needs of AA BCSs, interventions must focus on the attitudes, practices, and beliefs of this population (Davis et al., 2005). Few, however, have adopted this approach (Stolley et al., 2009).

This report described a process of engaging AA BCSs in developing an experiential nutrition education program. The outcomes of two 4-hour guided discussions were categorized as: solving barriers to accessing healthy foods in grocery stores and restaurants; linking behavior change to cultural values; and modifying traditional dishes to reduce calorie, fat, and sodium. Strategies for overcoming these barriers, including enhancing self-efficacy, promoting self-monitoring, and providing social support, were identified.

A similar approach of engaging AAs was utilized in Moving Forward (Stolley et al., 2009). A weight loss program designed for AA BCSs, its success was due, in part, to involvement of AA BCSs in the development of the intervention. Results, including high participant retention (87%), significant weight loss (mean=5.6 lbs [SD=6.5 lbs]), improved diet (a reduction in consumption of sweet and fatty foods and an increase in vegetable consumption by1.6 servings per day) and increased physical activity (median time spent in vigorous activity increased from 0 to 24 minutes per day), demonstrated the feasibility of recruiting, enrolling, and maintaining engagement of AA BCSs.

To enhance enrollment of AA BCSs in nutrition education programs aimed at reducing breast cancer recurrence, researchers should engage them in all stages of program development. Engagement of BCSs at the levels of program conceptualization and implementation may improve the effectiveness of the program.

## Figures and Tables

**Table 1:** Theory-Based Content of the Experiential Nutrition Education Sessions

Session	Title	Content	Theoretical Component
1	What’s in it for me?	Program requirements; diet-related cancer prevention guidelines	Outcome expectancy; self-efficacy; self-monitoring
2	Be SMART	Setting Specific, Measurable, Achievable, Realistic and Timely (SMART) goals	Goal setting; self-efficacy; self-worth; feedback
3	The proof is in the pudding	Reading labels; grocery shopping tour; guided discussion	Social support; feedback
4	In living color	Fruits; cooking demonstration; guided discussion	Social support; self-monitoring
5	Know your risk	Diet, body weight, and breast cancer recurrence	Social support, feedback, self-monitoring
6	What goes in …	Introduction to plate method; portion size	Self-monitoring; goal setting
7	Keeping score	Meal planning; eating during holidays and special occasions	Methods for self-monitoring, behavioral cues, identifying and overcoming barriers
8	How sweet it is	Looking for hidden sugar; cooking demonstration; guided discussion	Self-monitoring; problem solving; stimulus control
9	Lifestyle and breast cancer	Dietary intake, physical activity, tobacco and alcohol use, stress management	Self-efficacy; social support
10	What’s love got to do with it?	Promoting self-care	Self-efficacy; self-esteem; social support
11	Eat more for less	Portion control, energy-dense foods; grocery shopping tour; guided discussion	Self-efficacy; social support
12	Woman in the mirror	Red and processed meats; portion control; cooking demonstration	Self evaluation and assessment of progress toward SMART goal
13	Food for the soul	Transforming traditional dishes into healthier options	Problem solving
14	Taking charge of what’s around you	Controlling the environment; understanding your triggers and cues to action	Self-monitoring; problem solving; stimulus control
15	Eating on the run	Finding budget-friendly healthy foods in your area; grocery shopping tour; guided discussion	Self-efficacy; self-monitoring; social support
16	Stay in the game	Whole grans; cooking demonstration; guided discussion	Self-efficacy; self-monitoring; social support
17	Mind over matter	Stress management	Outcome expectancy; problem solving
18	Slim down	Weight control	Self-monitoring; outcome expectancy; problem solving
19	Restoration	Sleep, meditation, rest; grocery shopping tour; guided discussion	Self-efficacy; self-monitoring; social support
20	Fresh feast	Vegetables; cooking demonstration; guided discussion	Self-efficacy; self-monitoring; social support
21	It all works together	Whole goods and the holism of lifestyle change	Goal setting; problem solving; outcome expectancy
22	Get moving to better health	Physical activity benefits for breast cancer survivors	Self-monitoring; stimulus control
23	Nutrition and breast cancer	Super foods; grocery shopping tour; guided discussion	Self-efficacy; self-monitoring; social support
24	Looking back and moving forward	Celebration and strategies for maintenance; cooking demonstration; guided discussion	Outcome expectancy; self-efficacy; self-monitoring; social support
